# The Anti-Inflammatory Effect of a Combination of Five Compounds From Five Chinese Herbal Medicines Used in the Treatment of COPD

**DOI:** 10.3389/fphar.2021.709702

**Published:** 2021-10-27

**Authors:** Jiansheng Li, Peng Zhao, Yange Tian, Kangchen Li, Lanxi Zhang, Qingzhou Guan, Xiaofeng Mei, Yanqin Qin

**Affiliations:** ^1^ Henan Key Laboratory of Chinese Medicine for Respiratory Disease, Henan University of Chinese Medicine, Zhengzhou, China; ^2^ Collaborative Innovation Center for Chinese Medicine and Respiratory Diseases Co-constructed by Henan Province and Education Ministry of P.R. China, Zhengzhou, China; ^3^ Department of Respiratory Diseases, The First Affiliated Hospital of Henan University of Chinese Medicine, Zhengzhou, China; ^4^ Academy of Chinese Medical Sciences, Henan University of Chinese Medicine, Zhengzhou, China

**Keywords:** effective compound combination, chronic obstructive pulmonary disease, network pharmacology, inflammatory process, macrophage

## Abstract

Effective compound combination (ECC; i.e, 20-S-ginsenoside Rh1, astragaloside, icariin, nobiletin, and paeonol), derived from Chinese herbal medicine, significantly ameliorates chronic obstructive pulmonary disease (COPD) in rats; however, the underlying mechanisms of ECC remain largely unclear. In this study, network pharmacology analysis integrated with experimental validation was used to explore the therapeutic mechanisms of ECC against COPD. ECC targets and COPD genes and targets were identified from multiple databases, and then used for an analysis of protein–protein interaction (PPI) networks, Kyoto Encyclopedia of Genes and Genomes (KEGG) pathways, and biological functioning. BisoGenet was used to comprehensively analyze the hub-network. We validated the therapeutic effect and mechanisms of ECC both *in vivo* and *in vitro*. We identified 45 ECC targets, which were mainly related to inflammatory processes, such as the NOD-like and NF-kappa B signaling pathways, hematopoietic cell lineage, Th17 cell differentiation, cellular response to lipopolysaccharide, and interleukin-8 secretion. In addition, 1180 COPD genes and 70 COPD targets were identified as being involved in the biological functions associated with COPD development, such as cytokine–cytokine receptor interaction, the TNF signaling pathway, the mitogen-activated protein kinase (MAPK) signaling pathway, regulation of lymphocyte proliferation, and positive regulation of leukocyte migration. Integrative analysis of COPD genes and targets and ECC target networks revealed that 54 genes were mainly involved in the inflammatory process, such as IL-17 signaling, NF-kappa B signaling, innate immune response–activating signal transduction, and macrophage cell differentiation. Six targets (AR, ESR1, HNRNPA1, PAPR1, TP53, and VCAM1) contained in the hub-network and their four related compounds were obtained and recognized as the key molecules associated with the effects of ECC. Molecular docking validation demonstrated that four compounds could bind to six targets that interact with COPD genes. Finally, *in vivo* and *in vitro* experiments verified that ECC treatment ameliorated the symptoms of COPD in rats by improving their lung function, reducing pathological changes, and suppressing oxidative responses and pro-inflammatory cytokine secretion, while inhibiting inflammation in LPS-induced macrophages, which may be associated with NF-kappa B and MAPK signaling regulation. This study demonstrates the therapeutic mechanisms and effects of ECC on COPD *via* regulation of the underlying inflammatory process.

## Introduction

Chronic obstructive pulmonary disease (COPD) is a major respiratory disorder that is characterized by persistent airflow limitation and an abnormal inflammation response to noxious particles such as cigarette smoke (Wang et al., 2018). COPD is a leading cause of mortality, causing a significant economic and social burden in many countries worldwide (Zhou et al., 2019). However, to date, no drugs have been developed to specifically treat this disorder (Global Initiative for Choric Obstructive Lung Disease, 2020). The traditional Chinese medicine (TCM) has been shown to have a beneficial effect in the treatment of COPD patients. The Bufei Yishen formula (BYF; patent: ZL.201110117578.1) is a representative formula for the treatment of COPD patients with lung kidney qi deficiency syndrome, which is characterized by labored breathing, shortness of breath, fatigue, dizziness and tinnitus, frequent urination and nocturia, and weakness of the waist and knees (Li et al., 2012; Li et al., 2015). Our previous clinical study showed that COPD patients treated using BYF fared better, including a reduced frequency and duration of acute exacerbation COPD and an improved rate of decline in FVE1, than those treated with conventional Western medicine, while simultaneously demonstrating improved modified Medical Research Council dyspnea scale scores, six-minute walk distance (6MWD), and exercise tolerance (Li et al., 2021). In a previous study, the critical compounds derived from BYF were combined into a new effective compound combination (ECC) consisting of 20-S-ginsenoside Rh1, astragaloside IV, icariin, nobiletin, and paeonol (at a proportion of 25:5:100:4:6.25), which had a potential bioactive equivalence to BYF (Li et al., 2020). 20-S-Ginsenoside Rh1 and astragaloside IV have been reported to show anti-inflammatory, antioxidant, and immunomodulatory effects, and are widely used to treat inflammatory diseases (Ren et al., 2013; Chen et al., 2016; Tam et al., 2018). In addition, icariin, nobiletin, and paeonol have a wide range of pharmacological effects, such as anti-inflammatory, antitumor, immunomodulatory, and anti-lung injury effects (Liu et al., 2017; Li et al., 2018; Sun et al., 2018; Sun et al., 2020). However, the action mode and the underlying mechanisms of ECC for the treatment of COPD remain unclear, and it is difficult to gain a greater insight into these mechanisms while using traditional experimental approaches.

Network pharmacology was first constructed to identify systematic targets and explore the synergistic mechanism of multiple compounds. More recently, network pharmacology, which integrates target prediction, statistical analysis, network-based algorithms, and bioinformatics analysis with pharmacological validation, has been used to uncover the details of system interrelationships within “drug–target–pathway” diseases. Furthermore, we have previously applied system pharmacology to successfully predict the active compounds from BYF and their associated targets; their functions are mainly related to the activation of inflammatory and immune responses, and matrix metalloproteinases (Li et al., 2016). Nonetheless, the mechanisms of the five BFY effective compounds for treating COPD have not yet been demonstrated. Thus, we applied a network pharmacological analysis, based on both clinical and experimental data, to explore the potential mechanism of ECC for the treatment of COPD.

**FIGURE 1:**
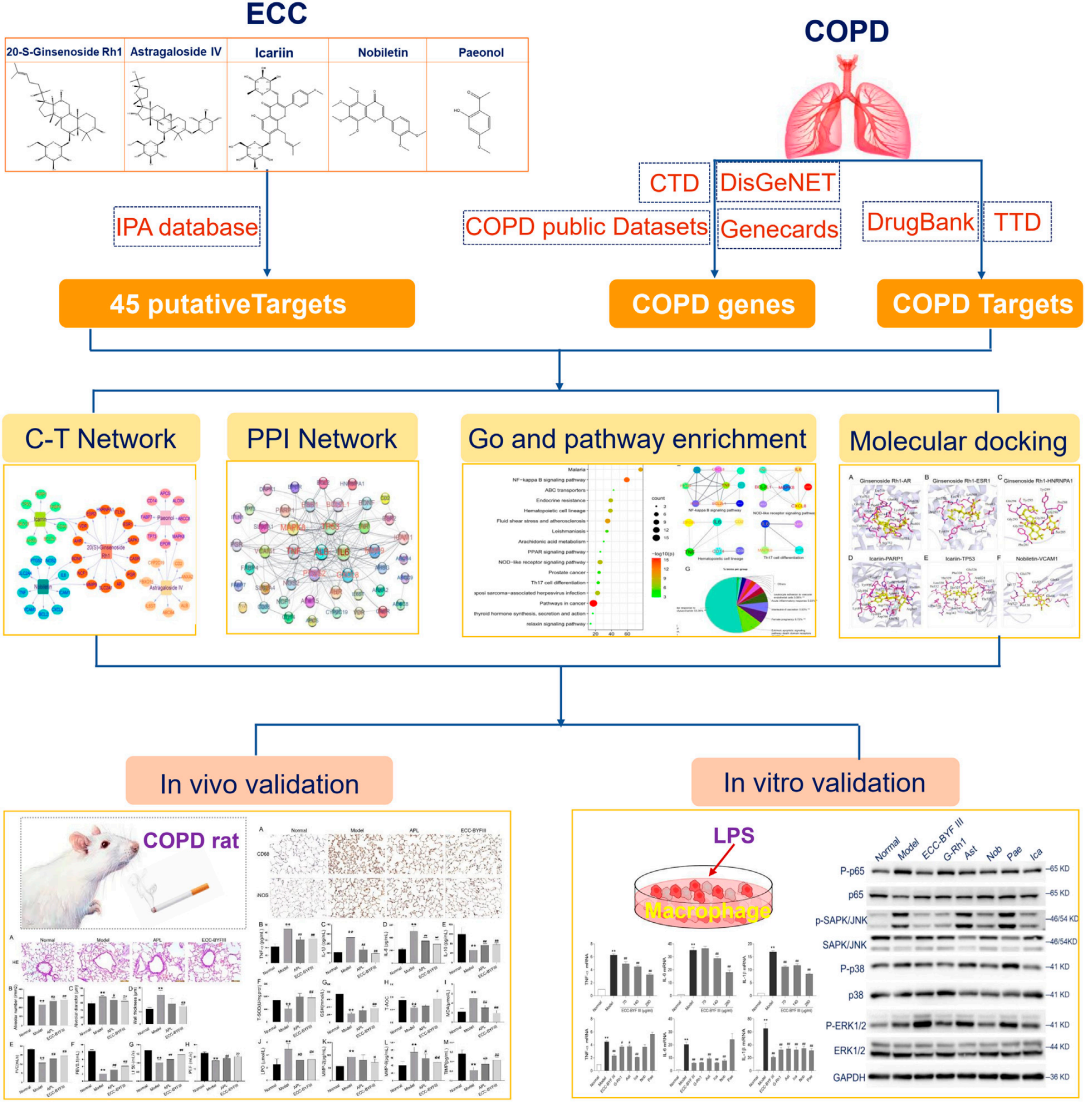
Integrative network pharmacology strategies to investigate the mechanism of ECC for the treatment of COPD.

In this work, we systematically explored the underlying mechanisms of ECC for the treatment of COPD using integrative network pharmacology strategies (Figure 1). In brief, we identified the putative targets of ECC from ingenuity pathway analysis (IPA), a list of COPD-related genes and targets from well-established databases. The network and pathway analyses were then employed to illustrate the molecular function, immune processes, and signaling regulation of ECC for the treatment of COPD. We further identified the important targets and pathways for ECC treatment, and used molecule docking to validate compound–target bindings. Consequently, we validated the therapeutic effect of ECC for the treatment of COPD in rats. *In vitro* experiments were also performed to validate the molecular mechanisms of the pharmacological action of ECC.

## Materials and Methods

### ECC–Target Prediction

ECC consists of 20-S-Ginsenoside Rh1 (PubChem CID: 12855920), astragaloside IV (PubChem CID: 13943297), icariin (PubChem CID: 5318997), nobiletin (PubChem CID: 72344), and paeonol (PubChem CID: 11092). The putative targets of these five compounds were identified using the following steps. First, we collected the putative targets of the compounds from the QIAGEN IPA, which was performed by the Beijing Computing Center. We then verified the reliability of the interactions of the compound–putative targets in the literature (Supplementary Table S1).

### COPD-Related Genes and Putative Targets

To identify COPD-related genes, we initially used the keyword “chronic obstructive pulmonary disease” to search in three databases: the Comparative Toxicogenomics Database (CTD; http://ctdbase.org/), the MalaCards (https://www.malacards.org/), and DisGeNET (http://www.disgenet.org/). We also identified COPD genes based on public data: a total of 137 human COPD samples and 119 normal lung tissue samples from the GSE38974, GSE57148, and GSE8581 datasets were obtained from the GEO database, as shown in Table 1. For the data assessed *via* sequence-based platforms, gene symbols were converted to Entrez gene IDs using the Biological Database Network (bioDBnet). Furthermore, differential gene expression analysis was performed using the significance analysis of microarrays algorithm (Tusher et al., 2001) and the Wilcoxon rank-sum test for array- and sequence-based data, respectively. A total of 422 genes, including 80 upregulated and 342 downregulated genes, were considered to be COPD-related genes and included in the subsequent analysis. The potential genes were screened according to their scores in the respective databases; when repeated at least three times in the four databases, they were considered COPD-related genes. COPD-associated targets were identified from the Therapeutic Target Database (http://db.idrblab.net/ttd/) and DrugBank (https://go.drugbank.com/) (Supplementary Table S2).

**TABLE 1:** Human COPD data used in this study.

	Platform	Normal	COPD
GSE38974	Agilent GPL4133	9	23
GSE8581	Affymetrix GPL570	19	16
GSE57148^a^	Illumina GPL11154	91	98

a These samples were measured by the RNA-seq platform.

### Functional enrichment analysis

The Metascape analysis resource (https://metascape.org) was used for the KEGG signaling pathway enrichment analysis. Molecular function, immune system, and biological processes were analyzed using ClueGo and Cluepedia in Cytoscape 3.7.2.

### Network construction and analysis

The ECC compounds and targets were used to construct the ECC–target network using Cytoscape 3.7.2. Protein–protein interaction (PPI) data were obtained from STRING 11.0 (https://www.string-db.org/) and applied for the construction of the PPI network.

BisoGenet, a Cytoscape plugin, was used to search for the molecular interactions of ECC targets, COPD genes, or COPD targets from “SysBiomics,” thus constructing three networks within Cytoscape, respectively (Martin et al., 2010). Consequently, the networks of ECC targets, COPD genes, or COPD targets that had been constructed in BisoGenet were merged, and a subnetwork was extracted according to node topological features, including degree, betweenness centrality, and closeness centrality (≥ 2 x median values).

### Molecular Docking

We downloaded the 3D structures (SDF file) of the compounds from the PubChem website (https://pubchem.ncbi.nlm.nih.gov/), whereas the protein crystal structures were extracted from the Protein Data Bank (http://www.rcsb.org/pdb). PyMol 2.3.4 was used to remove the waters, hydrogens, ions, and original ligands from the proteins. Finally, the proteins and ligands were saved using the AutoDock Tools, and each compound was docked into the target proteins.

### Chemicals and Animals

*Klebsiella pneumoniae* (strain ID: 46,114) was obtained from the National Center for Medical Culture Collection (Beijing, China). Tobacco was purchased from the Henan Tobacco Industry (Hongqi Canal^®^ Filter tip cigarette; tobacco type, tar: 10 mg; nicotine content: 1.0 mg; carbon monoxide: 12 mg; Zhengzhou, China). Aminophylline (APL) was purchased from Shandong Xinhua Pharmaceutical (Shandong, China). Rat IL-6, IL-10, MMP-2, MMP-9, and the TIMP-1 ELISA kits were obtained from Boster Biological Engineering (Wuhan, China). Total superoxide dismutase (T-SOD), malondialdehyde (MDA), glutathione (GSH), total antioxidant capacity (T-AOC), and lipid peroxidation (LPO) were obtained from Elabscience Biotechnology (Guangdong, China). Antibodies for mouse p65, p-p65, JUN N-terminal kinase (JNK), p-JNK, p38, and p-p38 were obtained from Proteintech (Wuhan, China). Qiazol lysis reagent was obtained from Qiagen (Valencia, CA, United States). HiScriptⅡQ RT SuperMix for qPCR and SYBR Green Master Mix were obtained from Vazyme Biotech (Nanjing, China). Fifty Sprague–Dawley rats (25 males and 25 females; 200 ± 20 g) were purchased from Jinan Pengyue Experimental Animal (Jinan, China).

### ECC Compounds and Ratios

ECC was composed of 20-S-ginsenoside Rh1 (Cas, 63,223-86-9), astragaloside (Cas, 84,687-43-4), icariin (Cas, 489-32-7), nobiletin (Cas, 478-01-3), and paeonol (Cas, 552-41-0) (Table 2). These compounds were purchased from Chengdu Must Bio-Technology (Chengdu, China). A purity of greater than 99% was determined using high-performance liquid chromatography, which was provided by Chengdu Must Bio-Technology. The ratio of these compounds in ECC was as follows: 25 20-S-ginsenoside Rh1: 5 astragaloside: 100 icariin: 4 nobiletin: 6.25 paeonol.

**TABLE 2:** Plant source of five chemical compounds.

No.	Chemical compounds	CAS	Herbal drug	Latin scientific name	Plant part(s)
1	20-S-Ginsenoside Rh1	63223-86-9	Ginseng Radix et Rhizoma	*Panax ginseng* C.A. Mey	Radix et Rhizoma
2	Astragaloside IV	84687-43-4	Astragali Radix	*Astragalus tibetanus* Bunge	Radix
3	Icariin	489-32-7	Epimedii Folium	*Epimedium acuminatum* Franch	Folium
4	Nobiletin	Cas, 478-01-3	Citri Reticulatae Pericarpium	*Citrus sinensis* (L.) Osbeck	Pericarpium
5	Paeonol	552-41-0	Paeoniae Rubra Radix	*Paeonia anomala* L	Radix

### Animal Experiments

The COPD rat model was developed as previously described (Li et al., 2012). The cigarette smoke exposure system primarily consisted of a closed chamber with a cigarette smoke producer. The rats were exposed to smoke in this smoke exposure system from 1 to 12 weeks and were repeatedly intranasally inoculated with *K. pneumonia* from 1 to 8 weeks.

Rats with COPD were randomly split into three groups: model group, ECC group, and APL group, which were orally administered normal saline, ECC (5.5 mg/ kg/ d), and APL (54 mg/ kg/ d) from 13 weeks of age, respectively. The normal rats were also orally treated with saline. At week 20, rats were anesthetized and sacrificed to collect lung tissue, blood, and bronchoalveolar lavage fluid (BALF). All animal experiments were approved by the Experimental Animal Care and Ethics Committee of the First Affiliated Hospital, Henan University of Chinese Medicine.

### Pulmonary Function, Histopathology, Cytokine, MMP, and Oxidative Factor Analysis

Peak expiratory flow (PEF), forced expiratory volume (FEV), and forced vital capacity (FVC) were detected using computer-controlled unrestrained pulmonary function testing plethysmography (Buxco Inc., Wilmington, NC, United States). The left lower lobe was fixed in formalin, and then embedded in paraffin. Paraffin sections were stained with Mayer’s hematoxylin and 1% eosin (H&E staining) or CD68, iNOS.

Lung tissue levels of TNF-α, IL-1β in BALF, IL-6, IL-10, MMP-9, and MMP-12 were determined using ELISA kits, according to the manufacturer instructions. MDA, LPO, T-SOD, GSH, and T-AOC were also assessed using ELISA kits, according to the manufacturer instructions.

### Cell Culture and Treatment

A mouse macrophage MH-S cell line was purchased from Shanghai Zishi Bio-Technology (Shanghai, China). MH-S cells were cultured in RPMI 1640 medium (Solarbio Life Science), which contained 10% fetal bovine serum (FBS), 100 U/ mL penicillin, and 100 μg/ ml streptomycin in a humidified 95% air/5% CO_2_ incubator at 37°C.

MH-S cells were seeded into six-well plates and cultured overnight. Cells were treated with different concentrations of ECC or its compounds for 3 h, and then exposed to LPS (100 ng/ ml) for 6 or 12 h. The concentrations of the compounds were as follows: 20-S-ginsenoside Rh1 (G-Rh1, 50 μg/ ml, 78.26 μM), astragaloside IV (Ast, 10 μg/ ml, 12.74 μM), icariin (Ica, 200 μg/ ml, 295.55 μM), nobiletin (Nob, 8 μg/ ml, 19.88 μM), and paeonol (Pae, 12.5 μg/ ml, 75.22 μM).

### Real-Time Polymerase Chain Reaction Assay

Cells were lysed with Qiazol lysing buffer, and total RNA was extracted according to the manufacturer instructions. RNA was reverse-transcribed into cDNA, and then used for Q-PCR with HiScriptⅡQ RT SuperMix and SYBR Green Master Mix (Vazyme; Nanjing, China). The primer sequences of the target gene are shown in Table 3. Last, gene expression was calculated using the 2^∆∆^Ct method.

**TABLE 3:** Primers used in the cellular experiments.

Gene	Primer 5'→3′
TNF-α	Forward-CAGGCGGTGCCTATGTCTC
	Reverse-CGATCACCCCGAAGTTCAGTAG
IL-6	Forward-CTGCAAGAGACTTCCATCCAG
	Reverse-AGTGGTATAGACAGGTCTGTTGG
IL-1β	Forward-GAAATGCCACCTTTTGACAGTG
	Reverse-TGGATGCTCTCATCAGGACAG
GAPDH	Forward-AGGTCGGTGTGAACGGATTTG
	Reverse-GGGGTCGTTGATGGCAACA

### Western Blot Assay

After treatment with ECC and its compounds, macrophages were lysed with RIPA buffer in ice. Protein samples with equal concentrations were separated using 10% SDS-PAGE gel and electro-transferred to PVDF membranes. Membranes with proteins were blocked with 5% nonfat milk, and then incubated with primary and secondary antibodies. Detection was performed using the Bio-Rad Imaging System.

### Statistical Analysis

All values are expressed as means ± standard errors of the means. Statistical differences were assessed using a one-way analysis of variance, followed by a *post hoc* Tukey’s test. P values < 0.05 were considered statistically significant. All statistical analyses were performed using SPSS 22.0 (IBM Corporation, Armonk, NY, United States).

## Results

### Functional Analysis of ECC Targets

**FIGURE 2:**
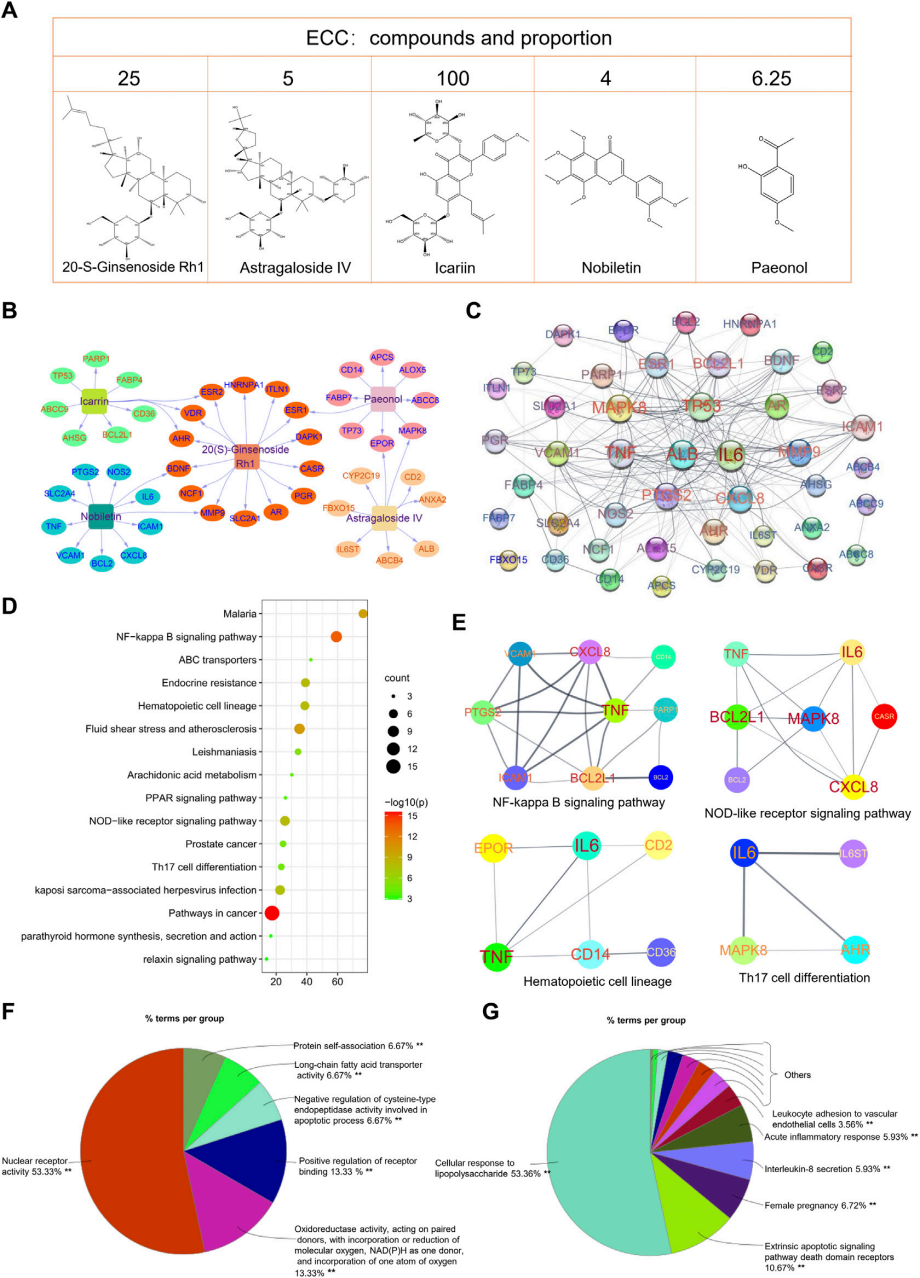
Bioinformatics analysis of ECC targets. **(A)** Compound–target network of ECC constructed using Cytoscape. **(B)** Protein–protein network of ECC targets constructed using STRING. The size of the label is proportional to its strength. **(C)** The top 20 enriched KEGG pathways of ECC targets constructed using Metascape. **(D, E)** Protein–protein interactions of the proteins contained in the top pathways. **(F)** Molecular function analysis and **(G)** biological process analysis for 45 targets constructed using ClueGo in Cytoscape.

The five compounds contained in ECC may exert a synergistic effect through multiple targets (Figure 2A). Thus, an accurate evaluation of the ECC targets was necessary. Based on the IPA database, we identified 45 targets for ECC, and then constructed the respective compound–target network (Figure 2B). The number of potential targets connected by 20-S-ginsenoside Rh1, astragaloside IV, icariin, nobiletin, and paeonol was 14, 9, 10, 11, and 9, respectively. As seen in Figure 2C, 42 of these 45 targets interacted with each other to construct a PPI network, in which inflammatory mediators and their regulators (e.g., TNF, IL-6, CXXL8, PTGS2, VCAM1, ICAM1, NOS2, and MAPK8) were contained.

The KEGG pathway enrichment analysis identified the signaling pathways associated with the 45 targets. As shown in Figure 2D, the top 20 KEGG pathways were markedly enriched with 45 targets, which were mainly involved in cancer, inflammatory, and immune system processes. Consequently, the regulation of immune and inflammation responses, including the NOD-like and NF-kappa B signaling pathways, hematopoietic cell lineage, Th17 cell differentiation, and PPAR signaling pathway, may be the major mechanism of ECC (Figure 2E). In addition, the molecular function and immune system process analysis demonstrated that the targets could regulate multiple biological functions, including oxidoreductase activity, cellular responses to lipopolysaccharide, interleukin-8 secretion, acute inflammatory responses, and leukocyte adhesion to vascular endothelial cells; these can effectively regulate inflammatory processes (Figures 2F,G).

### Functional Analysis of COPD-Associated Genes and Therapeutic Targets

**FIGURE 3:**
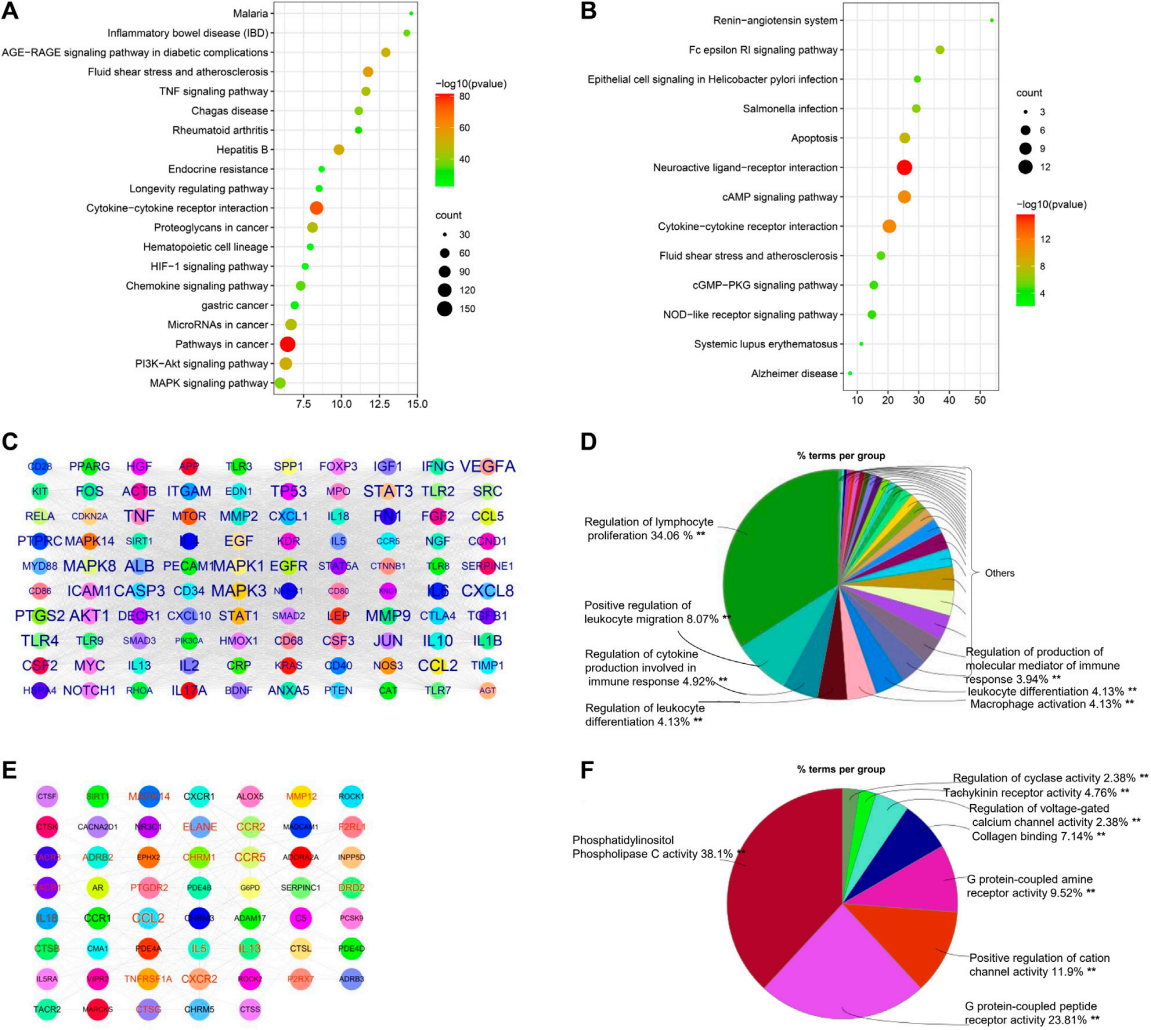
Bioinformatics analysis of COPD genes and targets. **(A)** Top 20 enriched KEGG pathways of COPD genes and **(B)** COPD targets, constructed using Metascape. **(C)** Protein–protein network of top 100 COPD genes constructed by Cytoscape. The size of the label is proportional to its strength. **(D)** Immune system process analysis of the top 100 COPD genes using ClueGo in Cytoscape. The size of the label is proportional to its strength. **(E)** Protein–protein network of COPD targets. **(F)** Immune system process analysis of COPD targets.

COPD pathogenesis is associated with innate and adaptive immune responses as a result of the inhalation of toxic particles and gases (Barnes, 2016); however, details regarding the mechanisms that dictate COPD pathogenesis remain unclear. We identified 1180 COPD-related genes from DisGeNET, CTD, GeneCard, and COPD public data. We performed KEGG pathway enrichment analysis using these COPD-related genes and noted that the top 20 KEGG pathways were markedly enriched with COPD genes, which could be categorized as related to inflammation regulation (cytokine–cytokine receptor interaction, TNF signaling pathway, chemokine signaling pathway, hematopoietic cell lineage, HIF-1 signaling pathway, mitogen-activated protein kinase (MAPK) signaling pathway, and PI3K-Akt signaling pathway), cancer, and infections, among other roles (Figure 3A).

To analyze the main COPD therapeutic targets and their mechanisms, 70 therapeutic targets of COPD drugs were collected from therapeutic target databases. Pathway enrichment analysis suggested that the major functions of the therapeutic targets primarily involved the regulation of immune responses, such as cytokine–cytokine receptor interaction, the Fc epsilon RI signaling pathway, the NOD-like receptor signaling pathway, apoptosis, the cAMP signaling pathway, and the cGMP-PKG signaling pathway, but also included infections and other functions (Figure 3B).

As shown in Figure 3C, 100 hub genes (e.g., MAPK3, MAPK14, PTGS2, ICAM1, CXCL8, JUN, and IL17A) were extracted from a PPI network of 1180 COPD genes based on their degree value, and then their immune system processes were analyzed. As shown in Figure 3D, results indicated that the hub genes of COPD-related genes were mainly involved in the regulation of lymphocyte proliferation, positive regulation of leukocyte migration, and macrophage activation, among other roles. Similarly, COPD targets were used to construct PPI networks for an analysis of their immune system processes. The collected data suggested that the hub genes, including CCL2, IL13, MAPK14, and ALOX5, were mainly associated with phosphatidylinositol phospholipase C activity, G protein–coupled peptide receptor activity, and the positive regulation of cation channel activity, which are regulated by COPD drugs (Figures 3E,F).

### Intersection Analysis Among ECC Targets, COPD Targets, and Genes

**FIGURE 4:**
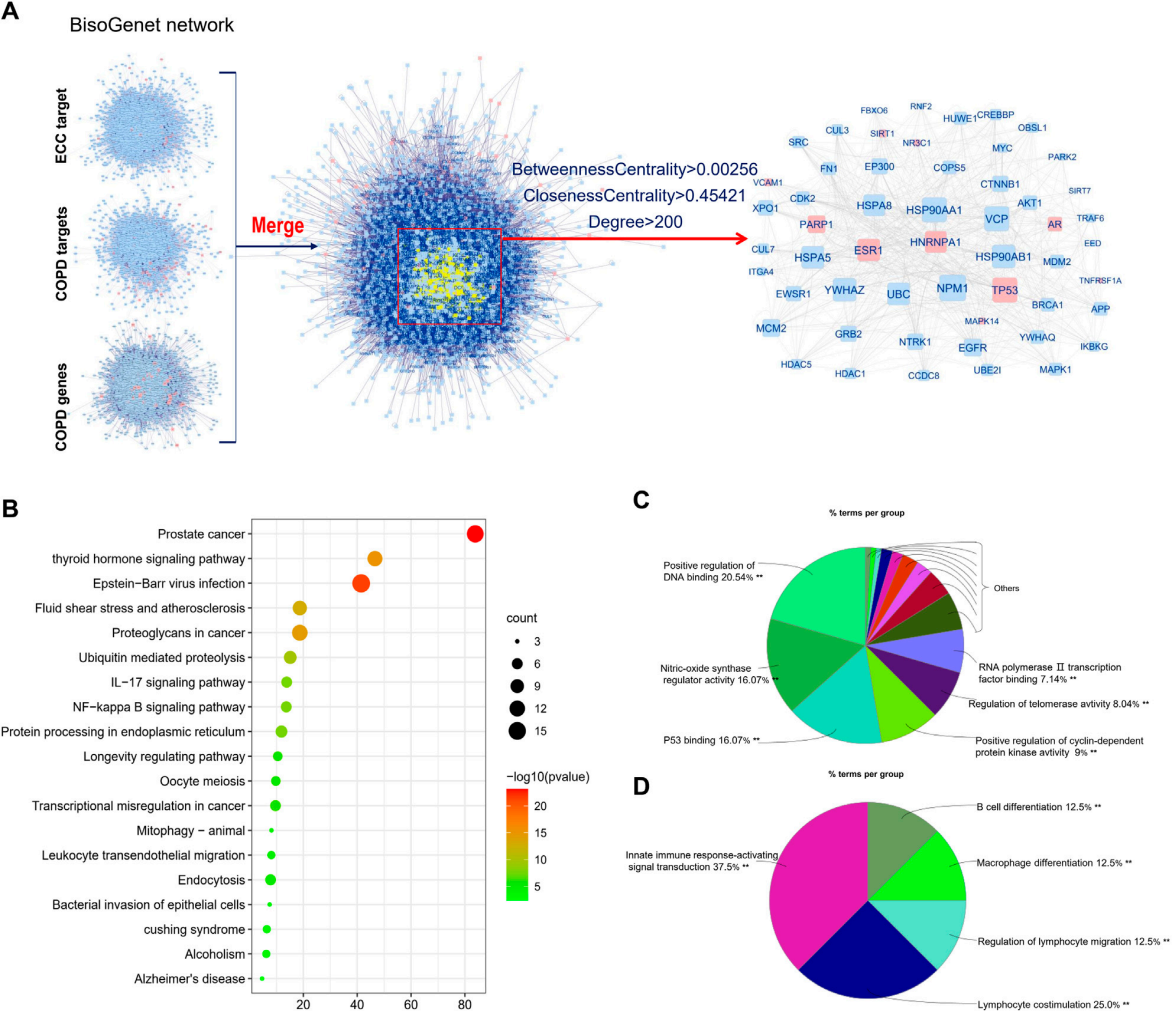
Integrative analysis of ECC targets, and COPD genes and targets. **(A)** Hub-network extracted from the merged network of ECC targets, COPD genes, and targets. **(B)** Top 20 enriched KEGG pathways of genes contained in the hub-network constructed using Metascape. **(C)** Molecular function and **(D)** immune system process analysis for the genes contained in the hub-network.

Similar to the above analysis, the ECC targets, COPD targets, and COPD genes were mainly associated with inflammatory responses, immune responses, and cancer, respectively; however, this analysis did not reveal the mechanisms of ECC with respect to the treatment of COPD. Consequently, we performed an intersection analysis among the ECC targets, COPD targets, and COPD genes; the obtained hub-network comprised 54 proteins that contained six ECC and three COPD–drug targets, respectively (Figure 4A). These 54 proteins were then analyzed to obtain a pathway from the KEGG database. As shown in Figure 4B, the top 20 KEGG signaling pathways were mainly involved in cancer, viral infections, and inflammatory processes, such as IL-17 and NF-kappa B signaling, leukocyte transendothelial migration, and the bacterial invasion of epithelial cells. To obtain a more comprehensive view of the therapeutic mechanisms of ECC, the 54 proteins were analyzed with respect to their molecular functions and immune system processes. Our findings revealed that the molecular functions of these proteins mainly involved the positive regulation of DNA binding and cyclin-dependent protein kinase activity, nitric-oxide synthase regulator activity, and p53 binding (Figure 4C). In addition, the immune system processes of these proteins were mainly associated with innate immune response–activating signal transduction, lymphocyte co-stimulation, regulation of lymphocyte migration, and macrophage and B-cell differentiation (Figure 4D). Therefore, our data suggest that ECC may have a critical impact as a treatment for COPD *via* intervention with the underlying inflammatory processes.

### Validation of ECC Targets by Molecular Docking

Among the 45 targets of the ECC compounds, six targets were included in the BisoGenet networks of the COPD genes and the COPD–drug’s targets. As a result, a subsequent molecular docking analysis was performed to verify whether the ECC compounds bind to these targets.

**FIGURE 5:**
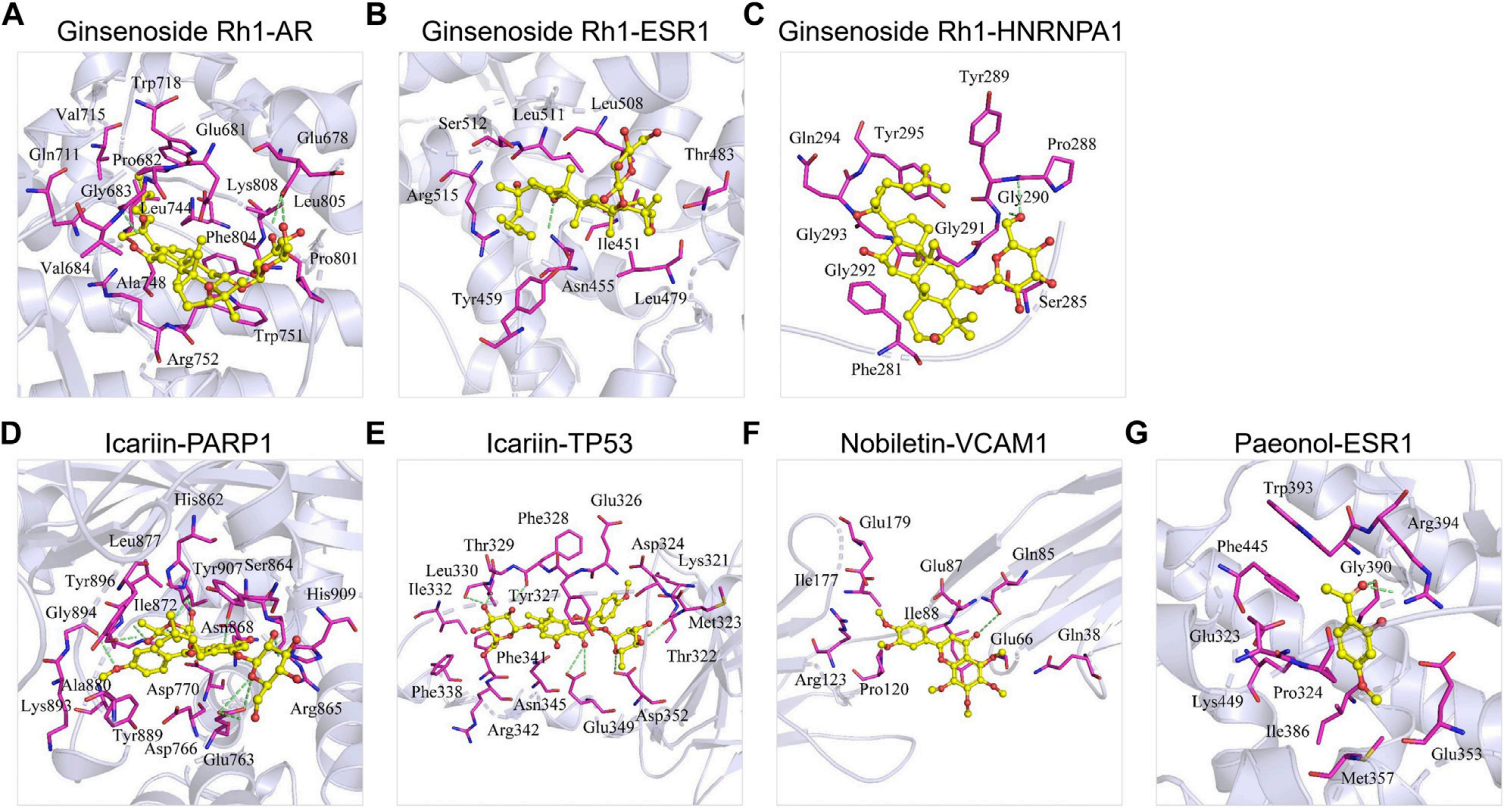
Molecular docking of ECC compounds with their targets. The binding poses of **(A)** ginsenoside Rh1 and AR, **(B)** ginsenoside Rh1 and ESR1, **(C)** ginsenoside Rh1 and HNRNPA1, **(D)** icariin and PARP1, **(E)** icariin and TP53, **(F)** nobiletin and VCAM1, and **(G)** paeonol and ESR1.

We collected the crystal structures of these six targets [i.e., AR (PDBID: 5V8Q), ESR1 (PDBID: 4XI3), HNRNPA1 (PDBID: 7BX7), PAPR1 (PDBID: 7KK2), TP53 (PDBID: 3Q05), and VCAM1 (PDBID: 1VSC)] from the Protein Data Bank, and obtained the chemical structures of these compounds from PubChem. The molecular docking score represented the affinity between the target protein and compounds. As shown in Figure 5; Table 4, the docking scores for the seven protein–ligand pairs were lower than −5 kcal/ mol, indicating that four compounds could bind to six targets. As shown in Figure 5A, the binding pattern between receptor protein AR and 20(S)-ginsenosideRh1 ligand, where the amino acid residues Glu678, Gly683, and Trp751 form hydrogen bonds with the ligand, and the amino acid residues Val715, Gln711, Pro682, Leu744, Val684, Ala748, Arg752, Trp751, Phe804, Pro801, and Leu805 form hydrophobic interactions with the ligand. As shown in Figure 5B, the amino acid residue Asn455 forms hydrogen bond with the 20(S)-ginsenosideRh1 ligand, and the amino acid residues Trp459, Arg515, Ser512, Leu511, Leu508, Thr483, Ile451, and Leu479 form hydrophobic interactions with the ligand. As shown in Figure 5C, the amino acid residues Gly290 and Pro288 form hydrogen bonds interactions with the ligand, and the amino acid residues Trp289, Gly291, Tyr295, Gln294, Gly293, Gly292, Phe281, and Ser285 form hydrophobic interactions with the ligand. As shown in Figure 5D, the amino acid residues Ser864, Asn868, His862, Arg878, Glu763, Ala880, Gly894, and His909 form hydrogen bonds with the ligand, and the amino acid residues Lys893, Trp896, Ile872, Leu877, His862, Tyr907, Ser864, Arg865, Asp770, Asp766, and Tyr889 form hydrophobic interactions with the ligand. As shown in Figure 5E, the amino acid residues Thr322, Asp352, Glu349, Phe328, and Leu330 form hydrogen bonds with the ligand, and the amino acid residues Ile332, Thr329, Tyr327, Glu326, Asp324, Lys321, Met323, Asn345, Phe341, and Phe338 form hydrophobic interactions with the ligand. As shown in Figure 5F, the amino acid residues Gln85 form hydrogen bonds with the ligand, and the amino acid residues Glu179, Ile177, Arg123, Pro120, Ile88, Glu87, Glu66, and Gln38 form hydrophobic interactions with the ligand. As shown in 5G, the amino acid residues Gly390 and Arg394 form hydrogen bonds with ligand small molecules, and the amino acid residues Trp393, Phe445, Glu323, Lys449, Pro324, Ile386, Met357, and Glu353 form hydrophobic interactions with the ligand. Thus, the ECC compounds may target these six proteins to regulate the activation of COPD genes and targets.

**TABLE 4:** Docking scores between ECC targets and compounds (kcal/mol).

Target compound	AR	ESR1	HNRNPA1	PAPR1	TP53	VCAM1
20(S)-GinsenosideRh1	−8.6	−6.3	−6.4	—	—	—
Icariin	—	—	—	−10	−7.6	—
Nobiletin	—	—	—	—	—	−6
Paeonol	—	−5.5	—	—	—	—

### ECC Alleviates the Severity of COPD in Rats

**FIGURE 6:**
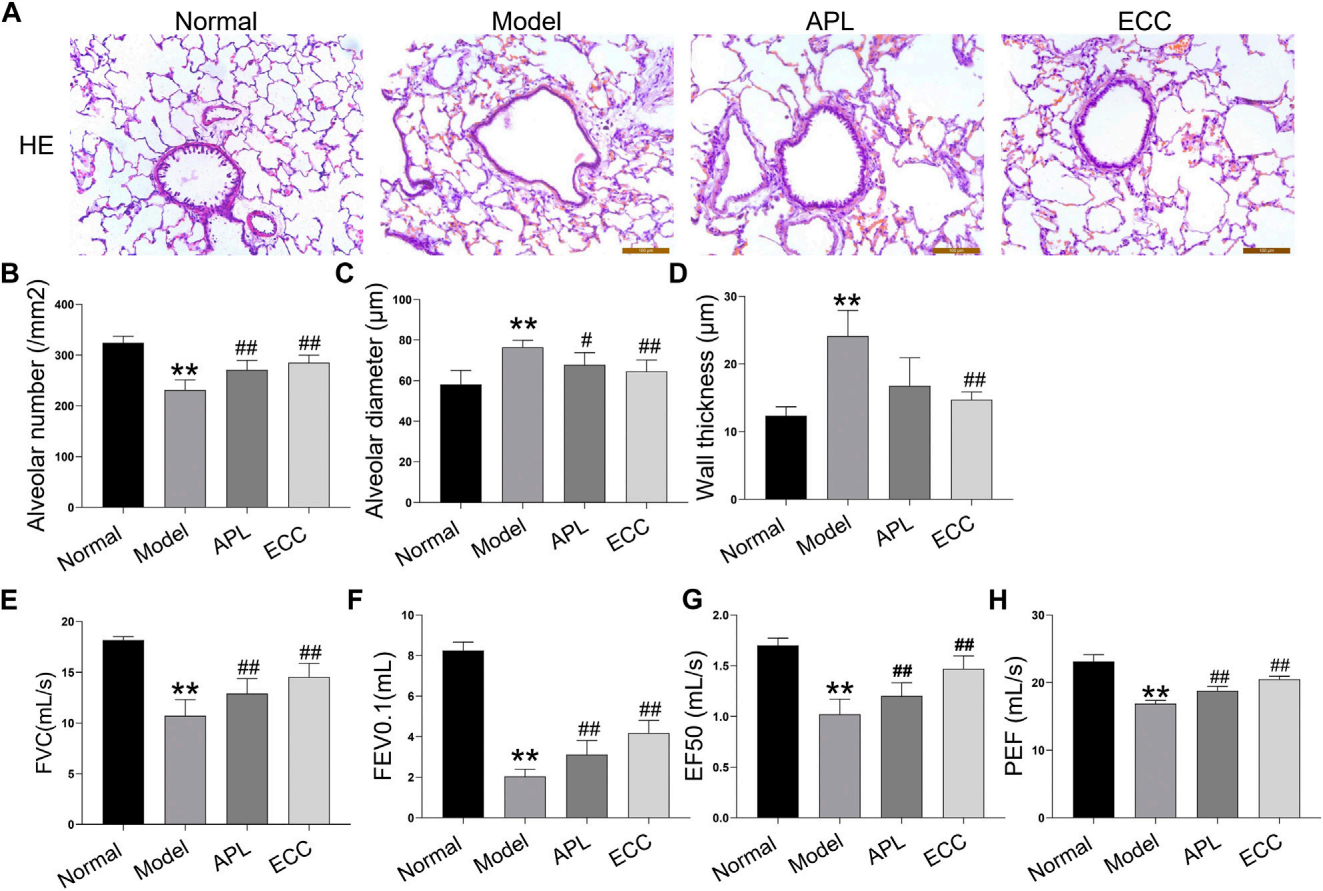
Effects of ECC on pathological changes and lung functioning in rats with COPD. COPD rats were treated with ECC (5.5 mg/ kg) and aminophylline (APL; 54 mg/ kg) for 8 weeks (weeks 12–20). **(A)** H&E staining (magnification, × 100), **(B)** alveolar number, **(C)** alveolar diameter, **(D)** wall thickness, **(E)** FVC, **(F)** FEV at 0.1 s, **(G)** peak expiratory flow, and expiratory flow at 50% tidal volume (EF50); and **(H)** PEF were assessed at week 20. All data are presented as the mean ± SEM (*n* = 6 for each group) *vs*. normal, **p < 0.01; *vs*. model, ^#^ *p* < 0.05, ^##^ *p* < 0.01.

To verify the therapeutic effect of ECC on COPD, we established a COPD rat model based on the simultaneous exposure of cigarette smoke and *Klebsiella pneumonia*. The rats with COPD were treated with ECC and APL on a daily basis for eight consecutive weeks, as opposed to the positive control. As shown in Figures 6A–D, hematoxylin and eosin (H&E) staining analysis of lung tissues demonstrated that ECC can increase the alveolar number while decreasing the alveolar diameter and wall thickness. There was a decrease in the pulmonary functioning of COPD rats, indicating a distinct airflow limitation. In contrast, ECC and APL treatment resulted in an increase in FVC, FEV0.1, EF50, and PEF (Figures 6E–H). Thus, our data suggest that daily treatment with ECC can attenuate the progression and severity of COPD.

### ECC Inhibits the Inflammatory Response, Oxidative Stress, and Protease Imbalance *In Vivo*

**FIGURE 7:**
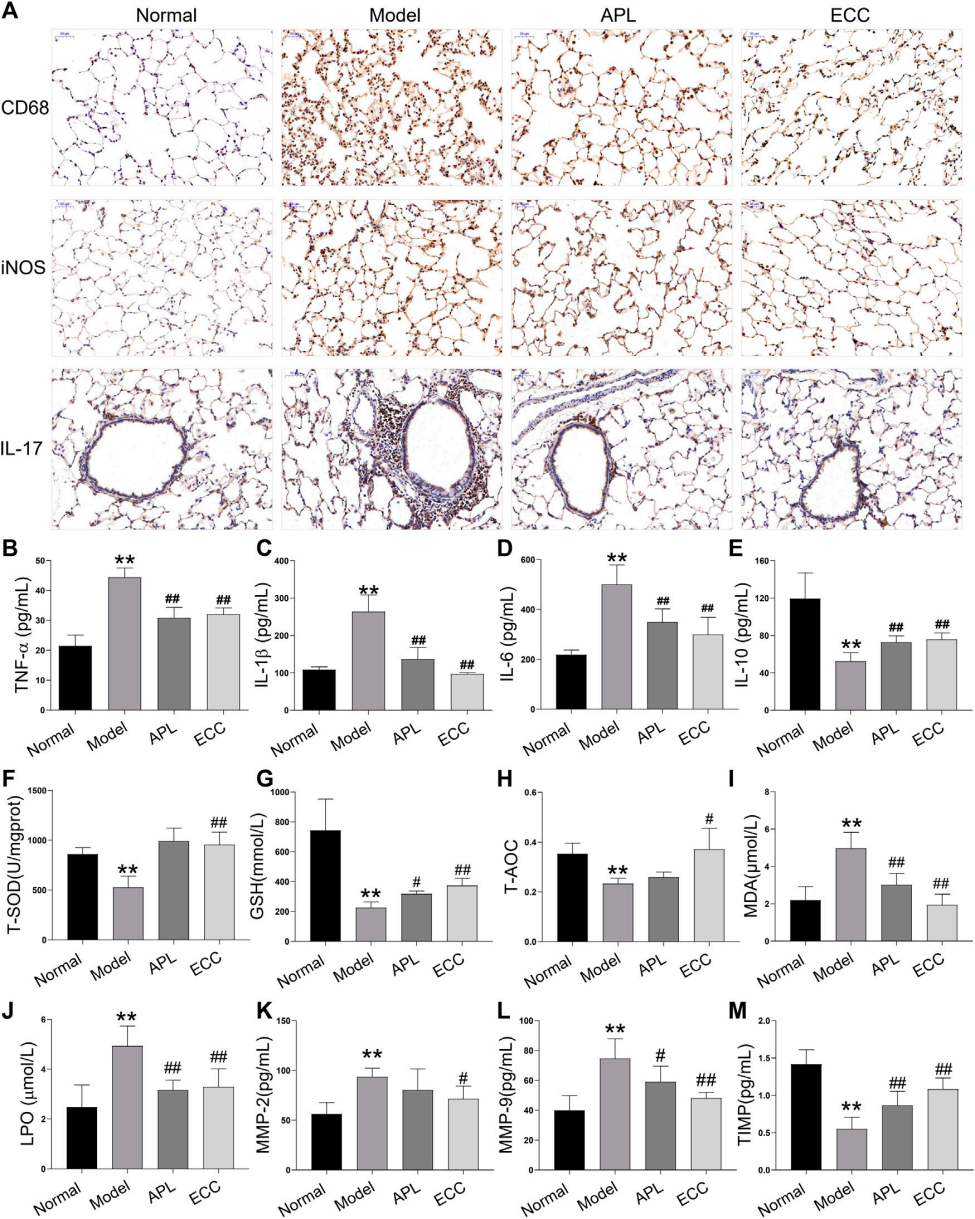
Effect of ECC on inflammation, oxidative stress, and proteinase/anti-proteinase in rats with COPD. Rats with COPD were treated with ECC (5.5 mg/ kg) and aminophylline (APL; 54 mg/ kg) for 8 weeks (weeks 12–20). **(A)** Macrophage marker, CD68, and iNOS were tested in lung tissue *via* immunohistochemistry. Levels of **(B)** TNF-α, **(C)** IL-1β, **(D)** IL6, and **(E)** IL-10 cytokines were detected using ELISA. Serum levels of **(F)** T-SOD, **(G)** GSH, **(H)** T-AOC, **(I)** MDA, and **(J)** LPO were detected using kits. In lung tissue, **(K)** MMP-2, **(L)** MMP-9, and **(M)** TIMP-1 were detected using ELISA. All data are presented as the mean ± SEM (*n* = 6 for each group) *vs*. normal, **p < 0.01, *vs*. model, ^#^ *p* < 0.05, ^##^ *p* < 0.01.

COPD is associated with systemic inflammation, which predominantly affects the airways and lung parenchyma (Hogg, 2009). Inflammation increases further in acute exacerbations of COPD, which represent a sudden worsening in airway functioning and respiratory symptoms (Bafadhel et al., 2011). Based on the above analysis, the ECC targets and COPD genes were mainly associated with inflammatory processes. Thus, we examined the inhibitory effect of ECC on inflammatory responses in rats with COPD. In the lung tissue of these rats, the macrophage number and pro-inflammatory cytokines, including IL-17, TNF-α, IL-1β, and IL-6, were all increased, whereas IL-10 was decreased. Therefore, ECC may reduce the inflammatory changes induced in rats with COPD (Figures 7A–E).

Noxious particles expose airways and lung parenchyma to reactive oxygen species, resulting in oxidative stress, which in turn causes injury and subsequent inflammation (Wiegman et al., 2020). Oxidative stress is a critical predisposing factor in COPD pathogenesis. Several markers of oxidative stress, such as MDA and LPO, have consistently been shown to be higher in COPD patients, whereas many antioxidant markers are lower (Singh et al., 2017). Thus, we assessed the levels of antioxidant markers, including T-SOD, GSH, and T-AOC, and oxidative stress markers, including MDA and LPO. In COPD rats, antioxidant marker levels were decreased, whereas oxidative stress markers were increased. Consequently, ECC treatment appears to markedly inhibit these serum changes (Figures 7F–J).

Emphysema, the critical COPD pathogenesis, is mainly induced by protease and antiprotease imbalances that result in pulmonary parenchymal damage. Inflammatory cells, such as macrophages and neutrophils, can produce a variety of proteases, such as matrix metalloproteinases (MMPs), neutrophil elastase, and cathepsins (Haq et al., 2011). Here, we tested the levels of MMP-2, -9, and tissue inhibitor of metalloproteinase (TIMP) 1, which are closely related to lung injury and airway remodeling. Our findings reveal that MMP-2 and -9 levels were increased in COPD lung tissue, TIMP-1 levels were decreased, and ECC suppressed both changes (Figures 7K–M).

Altogether, daily treatment with ECC may alleviate COPD *via* reducing the subsequent inflammatory responses, oxidative stress, and protease–antiprotease imbalance.

### ECC and Its Compounds Exhibited an Anti-Inflammatory Effect in LPS-Treated Macrophages

**FIGURE 8:**
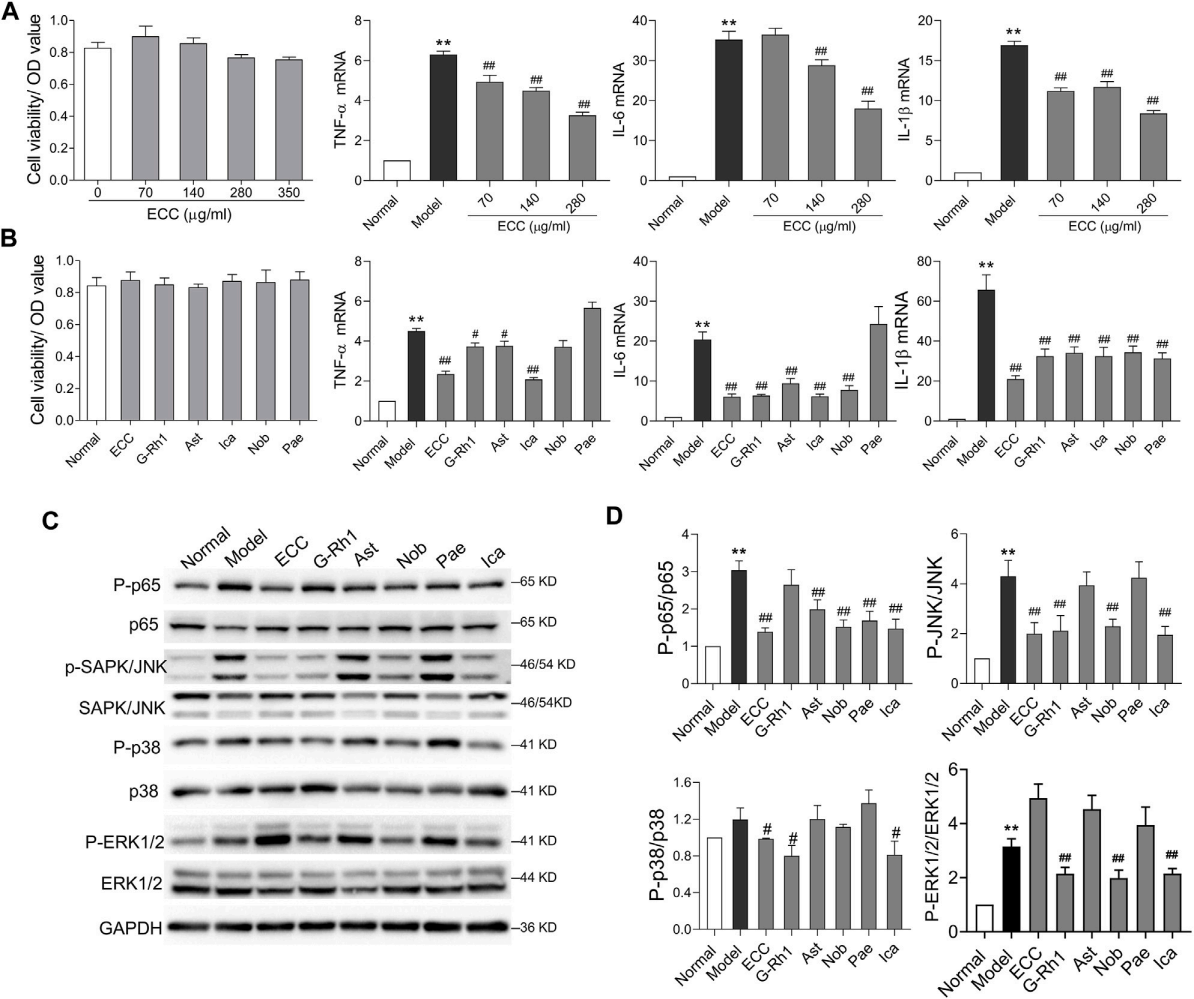
ECC suppressed cytokine levels and activation of NF-kB and MAPK signaling. Macrophages were treated with LPS (100 ng/ ml) and/or ECC (70, 140, and 280 μg/ ml), 20-S-ginsenoside Rh1 (G-Rh1, 78.26 μM), astragaloside IV (Ast, 12.74 μM), icariin (Ica, 295.55 μM), nobiletin (Nob, 19.88 μM), and paeonol (Pae, 75.22 μM) for 6 h. **(A)** Effect of ECC on cell viability and mRNA levels of TNF-α, IL-6, and IL-β. **(B)** Effect of ECC and compounds on cell viability and cytokine mRNA. **(C)** Detection of p-p65, p65, p-p38 (T180/Y182), p38, p-SAPK/JNK, SAPK/JNK, p-ERK1/2 (T202/185), and ERK1/2 *via* Western blotting. Data are presented as the mean ± SEM, *n* = 3. *vs*. normal **p < 0.01, *vs*. model ##*p* < 0.01, #*p* < 0.05. **(D)** Quantification of p-p65/p65, p-p38/p38, p-JNK/JNK, p-ERK1/2/ERK1/2.

Based on the above results, an inflammatory response, which is the critical cause of COPD progression, may be the major therapeutic mechanism involved with ECC treatment. Macrophages, the main contributors of cytokine secretion, are significantly increased in the lung tissues of rats with COPD, which play a critical role in orchestrating chronic inflammation (Barnes, 2014). Thus, we examined the effect of ECC and its compounds on the inflammatory response of LPS-treated macrophages. Notably, ECC decreased the levels of pro-inflammatory cytokines (TNF-α, IL-6, and IL-1β) in a dose-dependent manner and had no significant effect on cell viability (Figure 8A). In addition, the ECC compounds, including 20-S-ginsenoside Rh1, astragaloside IV, icariin, and nobiletin, downregulated the expression of TNF-α, IL-6, and IL-1β, and had no effect on cell viability; however, paeonol markedly decreased the levels of IL-1β only (Figure 8B).

According to the network pharmacology results, inflammatory processes, such as IL-17 and NF-kappa B signaling, were associated with the therapeutic mechanism of ECC. Furthermore, the IL-17 signaling pathway is mainly activated by the NF-kB and MAPK pathways, which include p38, extracellular signal–regulated kinase (ERK), and JNK (Le Rouzic et al., 2017). Thus, we assessed whether ECC and its compounds could regulate the activation of the NF-kB and MAPK pathways. As shown in Figures 8C,D, our results indicate that ECC decreased the levels of p-p65, p-JNK, and p-p38, but had no effect on p-ERK1/2. Five compounds were found to induce different effects on this signaling. For instance, 20-S-ginsenoside Rh1 significantly decreased the levels of p-JNK, p-p38, and p-ERK1/2, whereas astragaloside IV and paeonol only decreased p-p65 levels, and nobiletin and icariin suppressed the levels of p-p65, p-JNK, and p-ERK1/2; however, icariin also had an effect on p-p38 levels. These results suggest that ECC can inhibit inflammatory responses *via* suppression of the NF-kappa B and MAPK signaling pathways.

## Discussion

COPD is a serious health issue with a complex pathogenesis; the distinct lack of an effective therapeutic treatment for this disease leads to increased morbidity and mortality worldwide (Maigeng et al., 2019). BYF, a TCM for the treatment of COPD patients, contains thousands of compounds and targets from different proteins, and its efficacy is a result of an effective combination of compounds (Zhao et al., 2018). The critical effective compounds of BYF have been identified and combined into ECC, which has achieved bioactive equivalence with the original BYF (Li et al., 2021). ECC, composed of 20-S-ginsenoside Rh1, astragaloside, icariin, nobiletin, and paeonol, may treat COPD *via* a synergistic effect. In the current study, we integrated network pharmacology and *in vivo* and *in vitro* experiments to investigate the therapeutic mechanisms of ECC for the treatment of COPD.

Network pharmacology is a novel multidisciplinary method that integrates the immense volume of available information and allows for new discoveries. It usually predicts potential active compounds, protein targets, molecular function, and pathways using computational approaches, and validates these predictions using experimental approaches, including omics technologies and biological and pharmacological experiments. In consideration of ECC’s characteristics of unclear targets, and the complex biological activities and holistic regulation of COPD, we applied network pharmacology to investigate the therapeutic mechanisms of ECC for the treatment of COPD using both computational and experimental approaches (Li et al., 2014; Li and Zhang, 2013). First, we identified the putative targets for ECC compounds from IPA and the literature. 20-S-Ginsenoside Rh1 targets the highest number of proteins; thus, it may be the main active compound in ECC. In addition, PPI and pathway enrichment analysis suggested that the inflammatory response may be the critical biological process regulated by ECC compounds. For instance, the compounds’ hub targets, including IL6, TNF, MAPK8, PTGS2, CXCL8, and TP53, are closely related to inflammation. The top pathways, such as the NF-kappa B signaling pathway, Th17 cell differentiation, hematopoietic cell lineage, and the NOD-like receptor signaling pathway, were important in signaling for the regulation of the inflammatory response. Furthermore, the top immune system processes, including cellular responses to lipopolysaccharide, the extrinsic apoptotic signaling pathway *via* death domain receptors, interleukin-8 secretion, acute inflammatory response, and leukocyte adhesion to vascular endothelial cells, were the typical inflammatory processes. These results suggest that ECC targets multiple proteins, which collectively participate in the regulation of the inflammatory process.

Inflammation is a major biological process in the development of COPD (Barnes, 2016); however, it is unclear whether inflammatory response regulation is the underlying therapeutic mechanism of ECC in the treatment of COPD. Thus, we first collected 1180 COPD genes and 69 therapeutic targets from public databases, which were then used to analyze the molecular functions and pathways, thus uncovering the molecular mechanisms of COPD. The top pathways of the COPD genes and targets, such as the TNF signaling pathway, cytokine–cytokine receptor, MAPK signaling, the Fc epsilon RI signaling pathway, and epithelial cell signaling in helicobacter pylori infection, were mainly related to immune regulation. For instance, elevated levels of TNFα, and phosphorylation levels of p38 MAPK and ERK1/2, have been observed in COPD patients, and the inhibitors of p38 MAPK and TNFα have been shown to exhibit inhibitory effects on the inflammatory responses of COPD (Lemire et al., 2012; Zhang et al., 2019). Thus, the molecular mechanisms of inflammation may be critical in the subsequent pathogenesis of COPD.

ECC targets, COPD genes, and COPD targets were comprehensively analyzed with BisoGenet, from which the hub-network was extracted. Functional enrichment analysis showed that the genes contained in the hub-network were mainly associated with the IL-17 and NF-kappa B signaling pathways, nitric-oxide synthase regulator activity, innate immune response–activating signal transduction, and macrophage differentiation, which are closely related to the inflammatory response. IL-17 can activate the inflammatory transcription factor NF-κB and the MAPK pathways (i.e., p38, ERK, and JNK), which regulate the expression of pro-inflammatory cytokines in COPD (AudreeshbanerjeeKoziol-White and Jr, 2012; Schuliga, 2015; Le Rouzic et al., 2017). According to the network pharmacology analysis, PARP1, TP53, ESR1, HNRNPA1, VCAM1, and AR, which are contained in the hub PPI network of ECC targets, COPD genes, and targets, were considered important targets of ECC; in addition, molecular docking demonstrated that the compounds could bind to these targets, which are involved in the inflammatory response. Thus, our findings suggest that the inflammatory process is the critical therapeutic mechanism for both ECC and COPD progression.

We thus validated the anti-COPD and anti-inflammatory effect of ECC both *in vivo* and *in vitro*. Our results highlight that ECC treatment can ameliorate the symptoms of COPD in rats by improving lung function, reducing pathological changes, and suppressing the respective oxidative response and pro-inflammatory cytokine secretion. The IL-17 and NF-kappa B signaling pathways were the main inflammatory pathways related to both the ECC targets and COPD genes. In addition, the IL-17 signaling pathway was found to activate the inflammatory transcription factors to increase inflammatory genes, mainly through the regulation of NF-κB and MAPK (i.e., p38, ERK, and JNK; Li et al., 2019). Thus, we validated the anti-inflammatory and regulatory effects of ECC *via* NF-kappa B and MAPK signaling pathway effects in LPS-induced macrophages. Our results demonstrate that ECC can inhibit the inflammatory response by decreasing the expression of pro-inflammatory cytokines and suppressing the activation of MAPK (p38 and JNK) and NF-kappa B signaling. However, ECC can also increase the levels of p-ERK1/2 in LPS-induced macrophages. Overall, in this experiment, ECC treatment led to an alleviation of COPD, although an increase of p-ERK1/2 was found in LPS-induced macrophages. Additionally, 20-S-ginsenoside Rh1 (78.26 μM), astragaloside IV (12.74 μM), icariin (295.55 μM), nobiletin (19.88 μM), and paeonol (75.22 μM) exerted different effects on the inflammatory response in macrophages. More specifically, low concentrations of 20-S-ginsenoside Rh1, astragaloside IV, and nobiletin had the same anti-inflammatory effects as icariin. Thus, we speculate that as part of ECC, 20-S-ginsenoside Rh1 and astragaloside IV may play an important role in inhibiting inflammation. We previously evaluated the effect of ECC at three different doses on COPD rats, and found that ECC at middle dose could exert the best efficacy and presented with bioactive equivalence to the original BYF. Here, we only treated COPD rat with ECC at single dose, which showed significant beneficial effects on COPD rats. However, evaluation of therapeutic effect and underlying mechanisms of ECC at different doses is helpful to optimize the safe and effective use of the drug. Thus, the anti-COPD effect of ECC at different doses will be examined in the next work. Altogether, ECC appears to ameliorate the effects of COPD through suppression of the inflammatory response *via* MAPK and NF-kappa B signaling regulation.

## Conclusion

This work revealed the therapeutic mechanisms of ECC for the treatment of COPD using an integrative analysis of network pharmacology and *in vivo* and *in vitro* experiments. ECC appears to exhibit protective effects against COPD through the regulation of inflammatory responses *via* MAPK and NF-kappa B signaling.

## Data Availability

The original contributions presented in the study are included in the article/Supplementary Material; further inquiries can be directed to the corresponding author.
